# CD4 and MHCII phenotypic variability of peripheral blood monocytes in dogs

**DOI:** 10.1371/journal.pone.0219214

**Published:** 2019-07-03

**Authors:** Alicja Rzepecka, Magdalena Żmigrodzka, Olga Witkowska-Piłaszewicz, Anna Cywińska, Anna Winnicka

**Affiliations:** 1 Department of Pathology and Veterinary Diagnostics, Division of Animal Pathophysiology, Warsaw University of Life Sciences, Warsaw, Poland; 2 Department of Internal Diseases and Veterinary Diagnostics, Faculty of Veterinary Medicine and Animal Science, Poznań University of Life Sciences, Poznań, Poland; The Ohio State University, UNITED STATES

## Abstract

In humans and mice, the detailed phenotypic and functional characterization of peripheral blood monocytes allows for identification of three monocyte subsets. There are also evidences of monocyte phenotypic heterogeneity in other species, including cattle, sheep, pig and horse. However, little is known about such variability in dogs. The aim of the study was to determine whether and how peripheral blood monocytes of healthy dogs differ in the presence of MHCII and CD4 and in the basal production of reactive oxygen species (ROS). Three distinct subsets of CD11b^+^CD14^+^ monocytes were found in peripheral blood samples of healthy dogs, based on the variations in the density of MHCII and CD4 surface molecules: MHCII^+^CD4^–^ (Mo1), MHCII^+^CD4^+^ (Mo2) and MHCII^–^CD4^+^ (Mo3). The Mo2 and Mo3 were significantly lower in percentage than Mo1 but their basal ROS production was higher. Within the Mo2 and Mo3 subsets, the percentage of cells producing ROS was significantly higher comparing to cells lacking this activity. Canine peripheral blood monocytes vary in the expression of MHCII and CD4 and in the activity suggesting that cells within the three identified subsets carry out different functions. The higher production of ROS in non-activated cells within small subsets of Mo2 and Mo3 monocytes might indicate their immunomodulatory potential.

## Introduction

Phenotypic heterogeneity of monocytes in humans was firstly described in 1989 [1]. Currently, the classification of monocytes in human blood includes three subsets: classical CD14^++^CD16^–^, non-classical CD14^+^CD16^++^ and intermediate CD14^+^CD16^+^ [2]. Interestingly, each subset is specialized in certain activity, including the production of cytokines, reactive oxygen species (ROS) and phagocytosis [3]. They also seem to be differently involved in many types of human diseases, including coronary disease, asthma or tuberculosis [4, 5, 6]. CD14 (cluster of differentiation 14) is a common monocyte marker used to identify these cells not only in humans, but also in many other species, e.g. dog, cattle or horse [7, 8, 9]. CD16 (cluster of differentiation 16; also Fcγ receptor III) is primarily known as a marker of natural killer cells. It binds to antibodies and participates in signal transduction, which consequently stimulates cytotoxic activity of natural killer cells and leads to the transcription of genes encoding cytokines and other factors [10, 11]. Similar role of CD16 on monocytes has also been reported [12].

Monocyte subsets in mice are defined on the basis of variations in the expression of: Ly6C (lymphocyte antigen 6C), CX3CR1 (CX3 chemokine receptor 1), CCR2 (C-C motif chemokine receptor 2) and CD43 (cluster of differentiation 43), and similarly to those described in humans, they are identified as: Ly6C^++^CD43^+^ classical, Ly6C^++^CD43^++^ intermediate and Ly6C^+^CD43^++^ non-classical monocytes. There are also evidences of the presence of various monocyte subsets in other species, including cattle, sheep, pig and horse [13, 14, 15, 16]. Due to the differences in the presence of surface proteins and the availability of specific monoclonal antibodies, the identification of monocyte subsets in other species may differ, e.g. in rats—CD43 expression and variations in the expression of CD4 are taken into considerations, while in pigs CD14 and CD163 are examined [17, 18].

There are only few papers on phenotypic variations of monocytes in dogs. Gibbons et al. have recently reported that canine peripheral blood monocytes differ in the expression of CD14 and MHCII and are divided into three subsets, one of them lacking surface expression of CD14 [19]. Therefore, the authors suggested that these cells corresponded to non-classical monocytes. Interestingly, similarly to human and rat, a subset of canine monocytes also express CD4, but there are no specific data on the variability of this cells according to the presence of CD4 [20]. CD4 is known primarily as a T-cell differentiation antigen, however, it is also found on other cells, e.g. on human monocytes, macrophages and Langerhans cells [21, 22, 23, 24]. The presence and role of CD4 on monocytes in humans and other species seems underestimated and certainly has not been described well enough. In addition, dog is an exceptional species in terms of abundant expression of CD4 on peripheral blood neutrophils [25]. Cytometric identification of major leukocyte populations based on their morphological characteristics, represented by FSC (forward scatter channel) and SSC (side scatter channel;), is not sufficient for their precise separation, which is particularly important in evaluating the presence of various surface antigens. The cytometric identification of CD4 on peripheral blood monocytes in dogs is fraught with error due to the possibility of contamination of the monocyte region (relative to SSC) with neutrophils. Therefore, in our study, monocytes were identified based on the expression of CD14 and for their precise separation from neutrophils we have additionally used a CADO48a antibody specific to canine neutrophil antigens.

The production of ROS by monocytes is one of their prominent function associated with phagocytosis an intracellular killing. However, ROS are also involved in the regulation of cell singling cascades having an impact on cell proliferation and apoptosis. The effect depends on the amount of ROS. Produced at basal levels are required for cell singling, transduction and redox-dependent regulation [26]. For instance, ROS singling in MDSC (myeloid-derrived suppressor cells) allows them to modulate the function of other immune cells [27]. Moreover, increased production of ROS by monocytes may be associated with several inflammatory and pathologic conditions, e.g. hypertension or type 2 diabetes [28, 29]. It can be hypothesized that phenotypically different monocytes in dogs—similarly to humans, show a different basal ROS production, that determines their function and differencial ability to modulate immunity.

The objective of this study was to evaluate the phenotypic variability of CD14-positive monocytes in canine peripheral blood, in the context of the presence of CD4 and MHCII surface molecules as well as their basal production of ROS.

## Materials and methods

### Animals and blood samples

Thirteen healthy dogs, presented for periodical health examination in veterinary clinic in Choszczno, Poland, were included in the study. The inclusion criteria were: no clinical signs of the disease on clinical examination, results of haematological tests within the normal range for canine species, no vaccination and treatment during at least two weeks before blood sampling. The dogs were 7 months to 8 years old (median—2 years). Six males (one neutered) and seven females. Six dogs were Border Collie, one Cavalier King Charles Spaniel, one mixed breed, two Boxers, two German Shepherds and one French Bulldog. Only excess blood, collected as a part of routine diagnostic procedures was used for the study. The blood collections was a part of non-experimental clinical veterinary examination consented by the owners of dogs, therefore, according to the European directive EU/2010/63 and local regulations regarding animal experiments, there was no need for the approval of Ethical Committee.

Haematological tests were performed prior to cytometric analysis in blood samples anticoagulated with dipotassium salt of ethylenediaminetetraacetic acid, using ABX Micros 60 haematology analyzer (Horiba). In addition, leukogram was assessed in blood smears stained with May-Grünwald Giemsa method. The haematological procedures were performed in the Division of Animal Pathophysiology, Department of Pathology and Veterinary Diagnostics, Warsaw University of Life Sciences.

### Immunophenotyping

Anticoagulated whole blood samples were transfered into flow cytometry tubes to obtain 1x10^6^ leukocytes in each. An adequate amount of blood was determined on the basis of the total number of white blood cells. Subsequently, leukocytes were labeled in whole blood using antibodies specific for canine antigens or with documented cross-reactivity **(Table 1).** The appropriate amount and concentration of each antibody has been determined empirically in order to obtain optimal labeling results. Controls included unlabeled cells, available isotype controls and/or FMO (fluorescence minus one) controls. All antibodies were conjugated with fluorochromes, except CADO48a (specific for neutrophil antigens) and anti-CD11b. The cells were incubated with antibodies **(Table 1, No. 1–5)** for 30 minutes at 4°C in the dark. Then, red blood cells were lysed by incubation with 2000 μl of lysing solution (BD) at room temperature for 15 minutes in the dark and centrifuged (5 min., 300 x g). Supernatant was removed and the cells were washed twice by adding 500 μl of the flow cytometry staining buffer (phosphate buffered saline + 1% bovine serum albumin) and centrifugation (5 min., 300 x g). Subsequently, the cells were suspended in 100 μl flow cytometry staining buffer and labeled with CADO48a antibody **(Table 1, No. 6)** and secondary rat anti-mouse IgG1:BV510 **(Table 1, No. 7).** The cells were than incubated for 30 minutes at 4°C in the dark and washed twice (5 min., 300 x g). Finally, the cells were resuspended in 200 μl of flow cytometric staining buffer and immediately introduced into cytometer (FACSCanto II, BD).

**Table 1:** Antibodies (Ab) used in the study.

No.	Antigen	Host species and target species	Clone	Isotype	Fluorochrome	Source
**1**	MHCII	rat anti-dog	YKIX334.2	IgG2a	FITC	AbD Serotec
**2**	CD4	rat anti-dog	YKIX302.9	IgG2a	RPE-Cy7	AbD Serotec
**3**	CD14	mouse anti-human*	TÜK4	IgG2a	Pacific Blue	AbD Serotec
**4**	CD11b	mouse anti-dog	CA16.3E10	IgG1	unconjugated	AbD Serotec
**5**	(secondary Ab)	rat anti-mouse IgG1	A85-1	IgG1	phycoerythrin	Becton Dickinson
**6**	neutrophil’s antigen	mouse anti-dog	CADO48a	IgG1	unconjugated	Monoclonal Antibody Center
**7**	(secondary Ab)	rat anti-mouse IgG1	X56	IgG1	BV510	Becton Dickinson

*documented cross-reactivity with canine antigens [7]

### ROS production analysis

The cells were isolated from fresh heparinized whole blood by density gradient centrifugation, maintaining sterile conditions. Histopaque 1077 (Sigma-Aldrich) was used for the separation of peripheral blood mononuclear cells (PBMC), according to the manufacturer recommendations: 3 ml of blood was layered on 3 ml of Histopaque 1077 in a sterile, V-bottom tubes and then centrifuged (400 x g) for 30 minutes at room temperature. Collected fraction of mononuclear cells was then washed twice with RPMI 1640 culture medium (Sigma-Aldrich), followed by centrifugation for 7 minutes at room temperature (300 x g) and resuspended in 1 ml of RPMI 1640. Next, 10 μl of 0.4% trypan blue (Sigma-Aldrich) was added to 10 μl of the cell suspension to determine cell density and viability using Thoma counting chamber. To assess the production of reactive oxygen species, only the samples with > 95% of alive cells were used.

The basal production of reactive oxygen species by isolated monocytes was measured using CellRox Deep Red reagent (ThermoFisher), which penetrates cell membranes and upon oxidation in the presence of ROS exhibit fluorescence (emission wave at 665 nm). The isolated cells were resuspended in RPMI 1640 medium and transferred to a 96 flat bottom well plate at a concentration of 4 x 10^5^/200 μl. Cells were then incubated with CellRox Deep Red in a final concentration of 5 μM, and incubated for 30 minutes at 37°C with a controlled atmosphere of CO_2_. Cells without CellRox Deep Red served as a control. After incubation, the cells were transferred to flow cytometry tubes and washed in 500 μl of phosphate buffered saline (PBS) (5 min., 300 x g).

**Fig 1:**
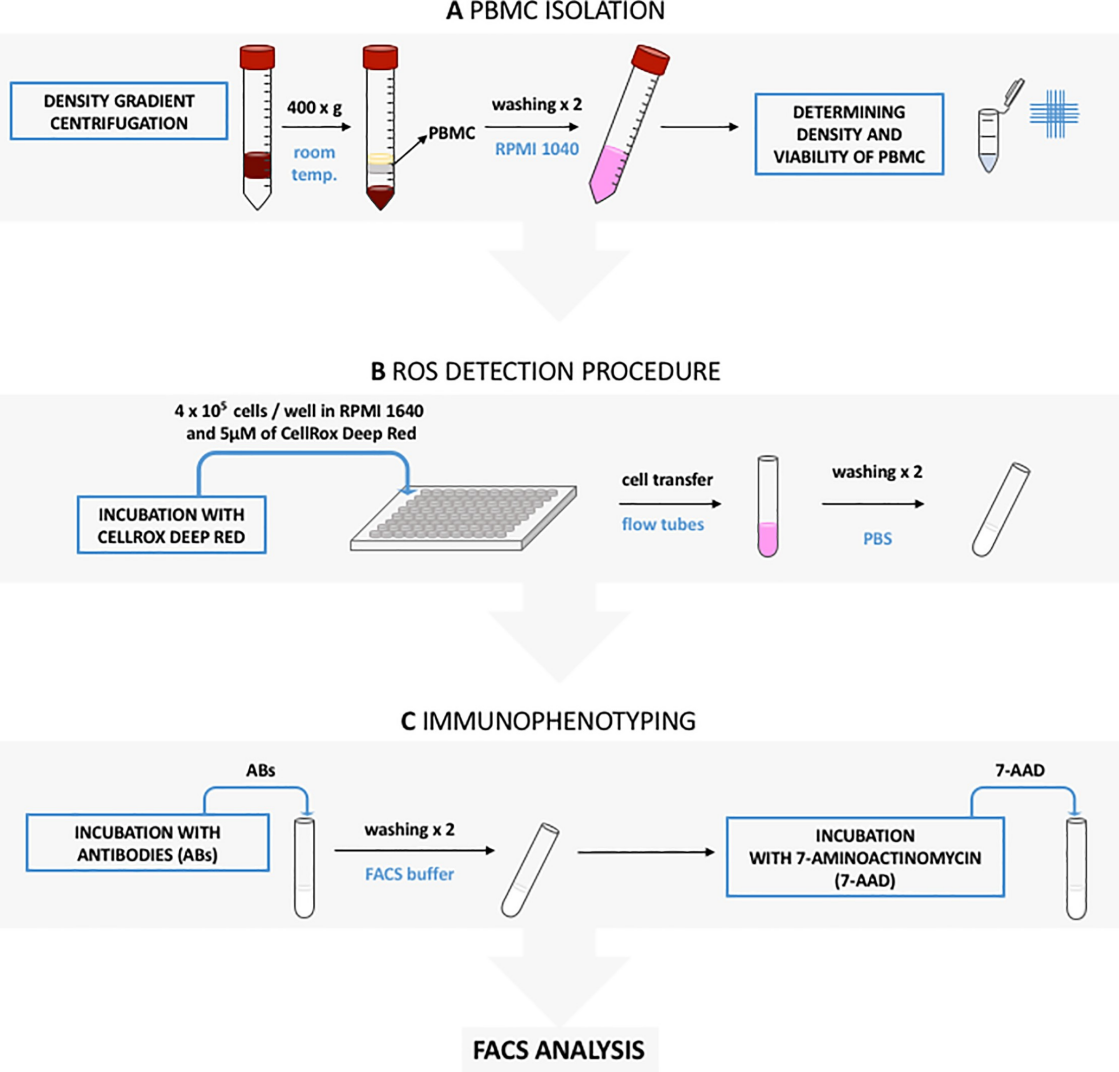
Figure depictng the procedure for detection of reactive oxygen species and cell immunophenotyping. (A) First, peripheral blood mononuclear cells (PMBC) were isolated from whole blood samples by density gradient centrifugation (400 x g in room temperature) and transfered into V-botom tubes. Collected fraction of PBMC was then washed twice with RPMI 1640 and resuspended in 1 ml of this culture medium. Next, 10 μl of 0.4% trypan blue was added to 10 μl of the cell suspension to determine cell density and viability using Thoma counting chamber. (B) In the second step the cells were resuspended in RPMI 1640, transfered into flat bottom 96 well plate in the number of 4 x 10^5^ cells/well and incubated with CellRox Deep Red in a final concentration of 5 μM. After the incubation step, cells were transfered into flow cytometric tubes and washed twice in phosphate buffered saline (PBS). Next, cells were resuspended in flow cytometry staining buffer and incubated with appropriate amount of antibodies (ABs). After two washing steps, cells were resuspended in flow cytometry staining buffer and incubated with the addition of 7-AAD (7-aminoactinomycin). After the incubation step flow cytometric (FACS) analysis was performed (C).

Subsequently, the cells were suspended in 100 μl of the flow cytometric staining buffer, labeled with antibodies listed in **Table 1 (no. 1–5)** and incubated 30 minutes at 4°C in the dark. After two-step washing, cells were suspended in 100 μl flow cytometry staining buffer and labeled with CADO48a antibody **(Table 1, no. 6)** and secondary rat anti-mouse IgG1:BV510 **(Table 1, no. 7)**, incubated as above, washed twice (5 min., 300 x g) and resuspended in 200 μl of the flow cytometric staining buffer. Finally, 5 μl of 7-aminoactinomycin (7-AAD; BD Pharmingen) was added in order to label dead cells. After 10 minutes, the cells were analyzed in the cytometer (FACSCanto II, BD). The process described above is illustrated in **Fig 1** in the order in which it was performed.

### Flow cytometry analysis

**Fig 2:**
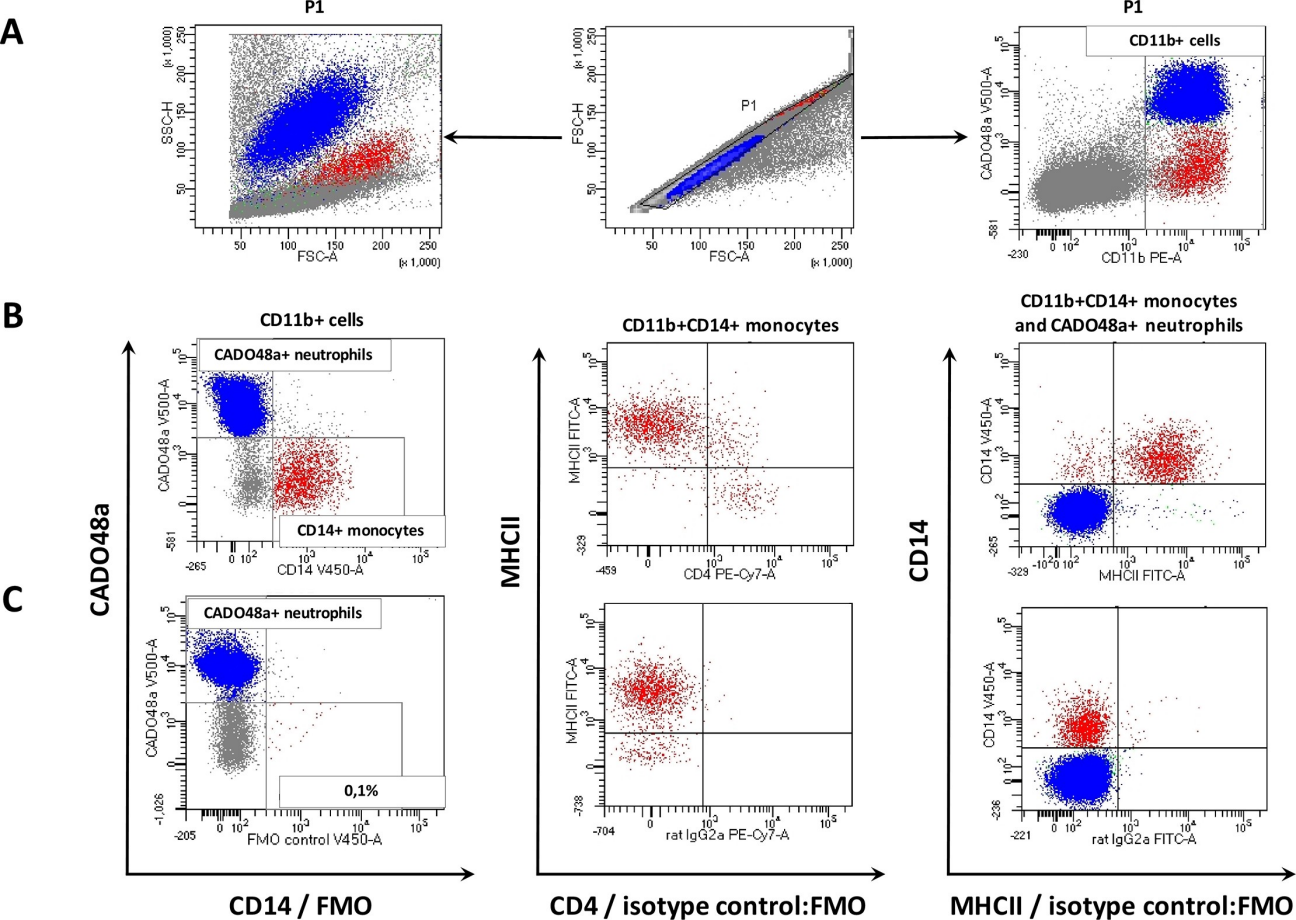
Gating strategy for identification of three monocyte subsets in peripheral blood of healthy dogs. (A) In order to eliminate doublets, single cells were gated (P1) on the FSC-H to FSC-A (middle) dot plot and depicted in a cytogram of size-to-granularity (left side) and dual-parameter dot plot—CADO48a:V500-A vs. CD11b:PE-A on which CD11b positive myeloid cells were gated (right side); (B) subsequently regions were set on CADO48a-possitive neutrophils (blue colour) and CD14-positive monocytes (red colour) (left side); CD11b^+^CD14^+^ monocytes were analysed by the expression of MHCII (FITC) and CD4 (PE-Cy7) (middle dot plot); the location of CADO48a-positive neutrophils comparing to CD14-positive monocytes was presented on the MHCII FITC-A vs. CD14 V450-A dual parameter dot plot (right side); (C) FMO control for CD14 allowed to set the region for positive cells; no specific staining for CD4 and MHCII was shown when using respective isotype controls together with other antibodies used for immunophenotyping (combined FMO and isotype controls).

Flow cytometric analysis was performed using a FACSCanto II flow cytometer and FACSDiva 7.0 software (BD). 30.000 cells of each sample were acquired. Prior to multi-color staining, the compensation was set using cells single-positive for each color. Doublets were removed from the analysis by setting the P1 gate on single cells on the FSC-area (FSC-A) vs. FSC-high (FSC-H) dot plot. The second region was set on myeloid cells (CD11b positive) on a double fluorescence dot plot: CD11b:PE-A vs. CADO48a:V500-A (**Fig 2A**). Then, CD14-positive monocyte population was gated and separated from CADO48a-positive neutrophils on CD14:V450-A (Pacific Blue) vs. CADO48a:V500-A dot plot. In the next step, CD11b^+^CD14^+^ CADO48a-negative cells were analysed for the surface expression of the MHCII and CD4. The locations of CADO48a^+^ neutrophils and CD14^+^ monocytes were visualized on CD14:V450-A (Pacific Blue) vs. MHCII:FITC dotplot **(Fig 2B**). For precise gates and quadrants setting, the following controls were used: FMO for CD14 and CD11b and isotype controls in combination with FMO for CD4 and MHCII **(Fig 2C).** For ROS detection, after eliminating the doublets and setting up the region for live cells (7-AAD negative) the gating strategy described above was used.

### Statistical analysis

Statistical analysis was undertaken using GraphPad Prism 6.0 software (GraphPad Software, La Jolla California USA). The results are presented as an arithmetic mean ± standard deviation. To determine the significance of differences in frequency between three populations and between the MFI (mean fluorescence intensity) on cells, the Friedman test with post-hoc test (Dunn’s test for multiple comparisons) was used. The significance of differences between two groups was determined using Wilcoxon test. In all tests, the value of p < 0.05 was considered significant.

## Results

### CD4 and MHCII expression on CD11b^+^CD14^+^ monocytes

**Fig 3:**
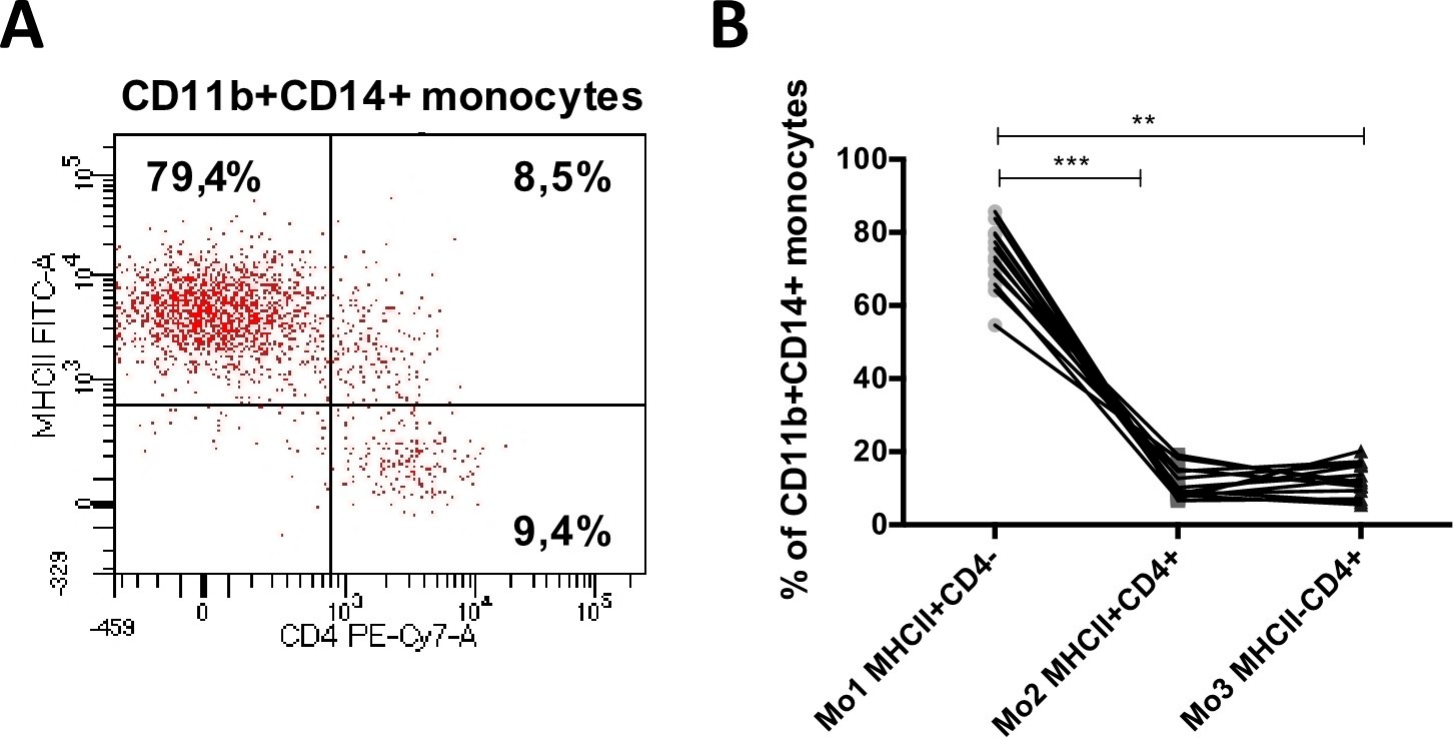
Monocyte subsets in peripheral blood of healthy dogs. (A) Representative dot plot depicting three CD11b^+^CD14^+^ monocyte subsets differing in the expression of MHCII (FITC-A) and CD4 (PE-Cy7-A): Mo1 MHCII^+^CD4^–^, Mo2 MHCII^+^CD4^+^ and Mo3 MHCII^–^CD4^+^. (B) The graph presenting their mean percentages in peripheral blood of healthy dogs. Data are presented as mean ± SD (n = 13); the significance was determined by ANOVA Friedman with Dunn’s post hoc tests (**p ≤ 0.01; ***p ≤ 0.001).

Immunophenotypic analysis allowed the identification of three subsets of CD11b^+^CD14^+^ monocytes differing in the expression of MHCII and CD4 molecules **(Fig 3A).** MHCII^+^CD4^–^ cells (Mo1) were predominant subset which comprised from 54.60% to 85.70% (73.07 ± 8.62) of all CD11b^+^CD14^+^ monocytes. The percentages of the other two subsets: MHCII^+^CD4^+^ (Mo2) and MHCII^–^CD4^+^ (Mo3) monocytes, were significantly lower (11.20 ± 4.28 and 12.12 ± 4.53, respectively) **(Fig 3B).**

### Morphological and phenotypical differences among monocyte subsets

**Fig 4:**
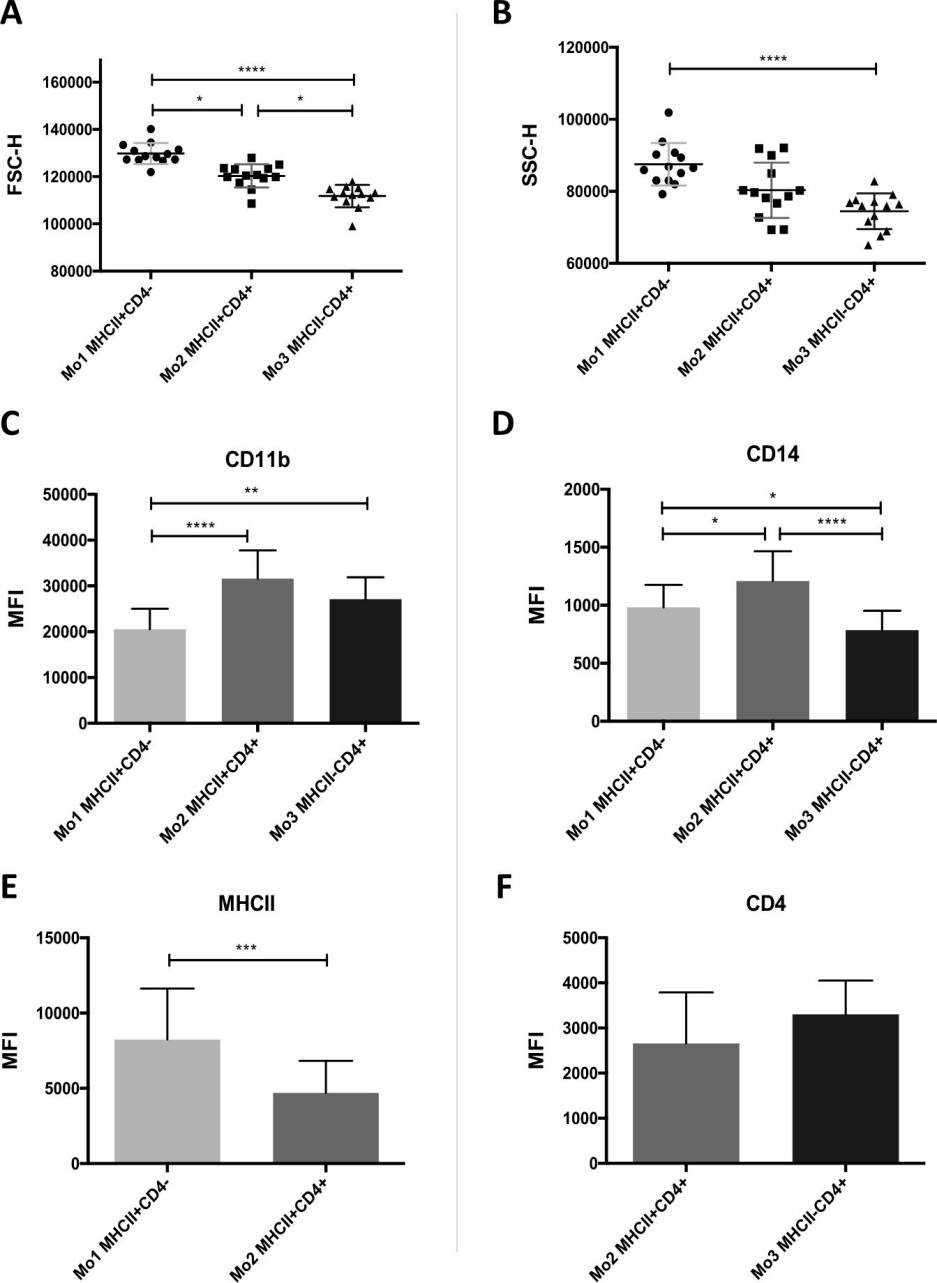
Morphological and phenotypical differences among monocyte subsets identified in peripheral blood of healthy dogs. Graphs presenting differences in (A) size and (B) granularity (according to FSC-H and SSC-H, respectively) among three CD11b^+^CD14^+^ monocyte subsets: Mo1 MHCII^+^CD4^–^, Mo2 MHCII^+^CD4^+^ and Mo3 MHCII^–^CD4^+^. Bar graphs of MFI (mean fluorescence intensity) of (C) CD11b, (D) CD14, (E) MHCII and (F) CD4 expressed on the cells within three monocyte subsets; for MHCII and CD4, MFI is presented for monocyte populations positive for these markers according to flow cytometry analysis. Data are presented as mean ± SD (n = 13); the significance was determined by ANOVA Friedman with Dunn’s post hoc tests (on A, B, C, D) and Wilcoxon test (on E, F) (*p < 0.05; **p ≤ 0.01; ***p ≤ 0.001; **** < 0.0001).

There were significant differences in cell size among three monocyte subsets **(Fig 4A)**: Mo1 were the largest cells–mean FSC-H of Mo1 was higher (129801 ± 4494) compared to Mo2 (120330 ± 4925, p = 0.0325) and Mo3 (111800 ± 4740; p < 0.0001). Mo3 was a subset of the smallest cells—the mean FSC-H Mo3 was lower compared to Mo2 (p = 0.0325). A similar tendency was also observed with respect to granularity (Mo1 87514 ± 5895, Mo2 80310 ± 7673, Mo3 74478±4970), however, significant difference was found only between Mo1 and Mo3 (p < 0.0001) **(Fig 4B)**.

For individual monocyte subsets, MFI and density of surface proteins (CD11b, CD14, MHCII and CD4), were also evaluated. Density of CD11b was significantly lower on Mo1 cells (20527 ± 4491) compared to the remaining monocyte groups—Mo2 (31609 ± 6161, p < 0.0001) and Mo3 (27096 ± 4813; p = 0.0051) **(Fig 4C)**. Mo2 showed a significantly higher density of CD14 (1210 ± 256) compared to Mo1 (983 ± 194, p = 0.0325) and Mo3 (785 ± 167; p <0.0001), and Mo1 expressed higher density of this marker compared to Mo3 (p = 0.0325) **(Fig 4D)**. Mo2 also showed significantly lower expression of MHCII (4693 ± 2139) compared to Mo1 (8248 ± 3382, p = 0.0005) **(Fig 4E)**. No significant differences were found in CD4 expression between CD4^+^ monocyte subsets: Mo2 and Mo3 (2659 ± 1132 and 3302 ± 750, respectively) **(Fig 4F)**.

### ROS production by monocytes

**Fig 5:**
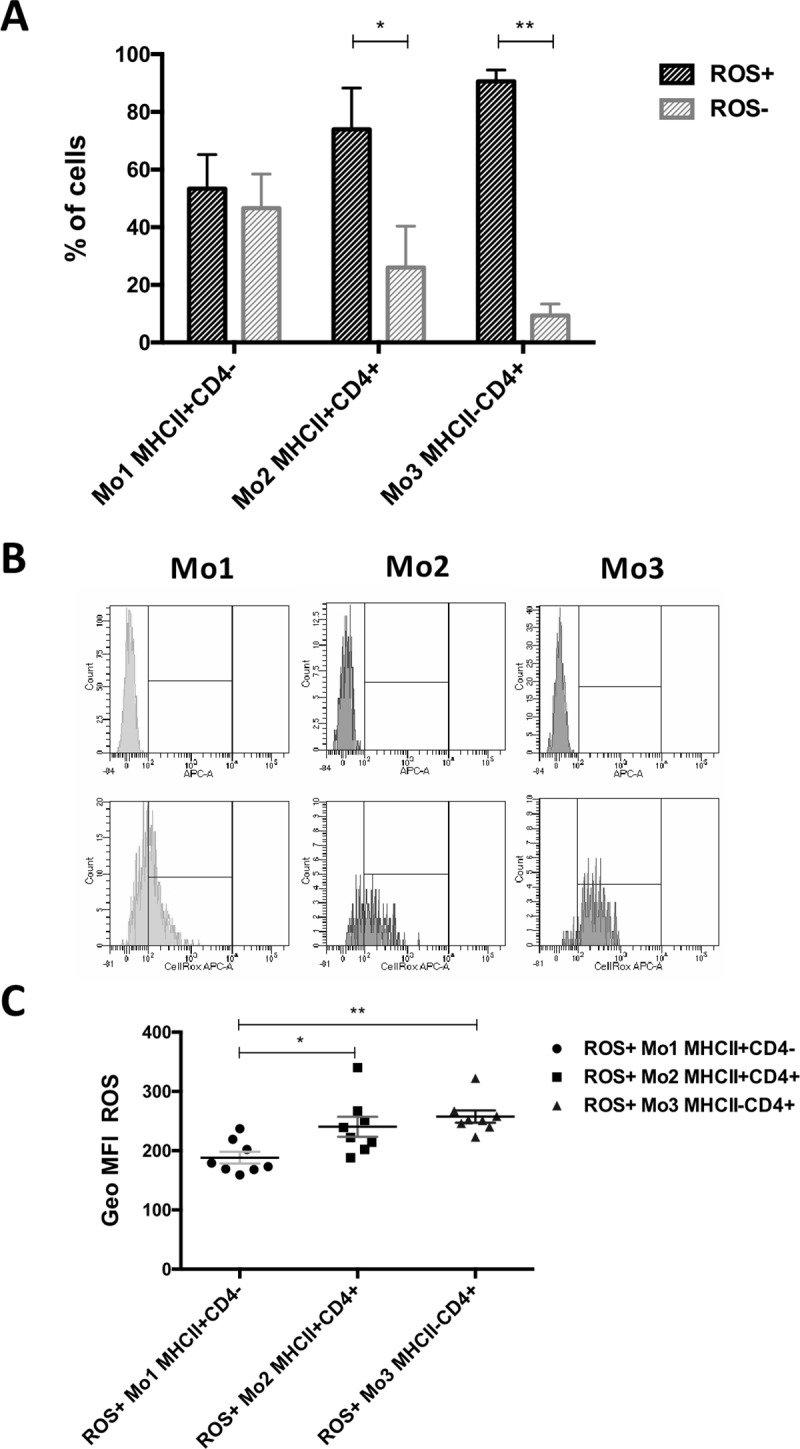
The basal production of reactive oxygen species by canine monocyte subsets. (A) The percentages of non-activated cells producing reactive oxygen species (ROS+) and non-producing reactive oxygen species (ROS-) within each monocyte subset: Mo1 MHCII^+^CD4^–^, Mo2 MHCII^+^CD4^+^ and Mo3 MHCII^–^CD4^+^; (B) representative fluorescence histograms depicting cytometrical analysis of ROS production by the cells. (C) Geo MFI (geometric mean fluorescence intensity, given by flow cytometer) of ROS for positive cells (producing ROS in non-activated state) from each monocyte subset. Data are presented as mean ± SD (n = 8); the significance was determined by Wilcoxon test (A) and ANOVA Friedman with Dunn’s post hoc tests (C) (*p < 0.05; **p ≤ 0.01).

The phenotyping of monocytes isolated in a density gradient allowed the selection of the same three cell subsets as phenotyped in whole blood: the most numerous—MHCII^+^CD4^–^ (Mo1; 67.84 ± 12.13) and two less numerous: MHCII^+^CD4^+^ (Mo2; 12.01 ± 4.52) and MHCII^–^CD4^+^ (Mo3, 18.54 ± 10.57). In each subset, some cells spontaneously produced reactive oxygen species. Cells producing reactive oxygen species (ROS+) represented the majority, compared to ROS-negative (ROS-) monocytes, among Mo2 (ROS+ 73.93 ± 14.33 vs. ROS- 26.08 ± 14.33; p = 0.0156) and Mo3 (ROS+ 90.59 ± 3.95 vs. ROS- 9.41 ± 3.95, p = 0.0078) cells. Within the Mo1 subset, on average, about half of the cells were ROS-negative (ROS+ 53.38 ± 11,81 vs. ROS- 46.63 ± 11.81) **(Fig 5A and 5B).** We have also found that ROS+ Mo1 produced significantly less ROS (188.30 ± 27.94) compared to ROS+ Mo2 (240.40 ± 47.81, p = 0.0374) and ROS+ Mo3 (257.40 ± 29.16, p = 0.0014) **(Fig 5C).** No significant differences were found in ROS production between Mo2 and Mo3.

### CD4 expression on CD11b^+^CADO48a^+^ neutrophils

Neutrophil labeling with CADO48a antibody was used for their separation from monocytes and to eliminate from the analysis. However, this strategy also allowed to observe the differential expression of the CD4 molecule on neutrophil surface. It was found that the vast majority of CD11b^+^CADO48a^+^ neutrophils expressed CD4 marker (92.55 ± 4.92) however, there was also a small population of CD4-negative neutrophils (7.45 ± 4.92). In comparison to CD4^+^ neutrophils, CD4^–^ cells showed significantly lower expression of CD11b (24048 ± 5768 vs. 18180 ± 5236, respectively, p = 0.0002) and CADO48a (10851 ± 4105 vs. 9456 ± 3234 respectively, p = 0.0005). According to FSC-H and SSC-H measurements, CD4^–^ neutrophils were also significantly smaller than CD4^+^ (84733 ± 5685 vs. 88785 ± 6087, p = 0.0061) and less granular (104030 ± 15540 vs. 128084 ± 8293; p = 0.0002) **(S1 Fig).**

## Discussion

In this paper, we have shown the phenotypic variability of canine CD14^+^ monocytes regarding the expression of MHCII and CD4 and basal ROS production. Our findings indicate a heterogeneity of these cells, similarly as described in other species. It should be emphasized that species differences prevent a strong assumption that the monocyte subsets identified in this work represent classical, intermediate or non-classical monocytes. The studies on monocyte phenotypic variation in dogs are at the initial stage. The only paper that describes the heterogeneity of these cells in dogs was published by Gibbons et al. [19]. The evaluation of CD14 and MHCII expression on CADO48a^–^CD5^–^CD21^–^ cells allowed the researchers to identify three monocyte subsets with the phenotypes: CD14^+^MHCII^+^, CD14^+^MHCII^–^, (described also in this paper) and, interestingly monocytes that do not express CD14 but positive to MHCII. Gibbons et al. therefore hypothesized that CD14^–^MHCII^+^ monocytes were equivalent to human non-classical monocytes, while CD14^+^MHCII^+^ represented intermediate and CD14^+^MHCII^−^was classical monocytes in the dog. It seems, however, that comparisons to human monocyte subsets with respect to the presence of MHCII, as proposed by Gibbons et al., should be done carefully because in humans this antigen, although not equally, is present on all monocytes. In addition, such division is not confirmed by the number of cells that constitute individual subsets of monocytes in humans. Leading researchers of monocyte subsets in humans look with a rising interest at analogous studies on monocytes conducted in various animal species [30]. They emphasize, that due to species differences and the inability to identify the same markers, direct comparisons still leave a lot of uncertainty.

The results we have presented provide a basis for future research on monocyte subsets in dogs. It would be valuable to evaluate the expression of CD16 on canine cells, however, it is currently not possible due to lack of specific antobodies. In subsequent experiments, it would also be necessary to determine the role of the CD4 antigen. Interesingly, in humans, CD16 is present on neutrophils and two monocyte subset, that provide a similar pattern to abundant expression of CD4 on majority of canine neutrophils and some monocytes. Perhaps CD4 has a similar role on the canine cells as CD16 in humans. Nevertheles, 65–90% of human CD14^+^ monocytes also express CD4 antigen, although at low density [31]. The significance of CD4 on human monocytes and macrophages has not been clearly described. In T lymphocytes CD4 is involved in response to antigen stimulation—it binds to a monomorphic MHC class II regions, which facilitates the binding of those cells to antigens even at their low affinity [32]. In addition, the cytoplasmic domain of CD4 poses a coreceptor for TCR (T-cell receptor), being involved in the stimulation of protein tyrosine kinase activity in stimulated T lymphocytes. This indicates a significant contribution of CD4 in cell activation, which is initiated by ligating the TCR [33, 34, 35, 36]. It has also been proven that blocking specific CD4 regions leads to the changes in cell activity dependent on this molecule [37]. Zhen et al. proved, that activation of CD4 (binding of CD4 with its ligand–MHC class II) molecule on human peripheral blood monocytes stimulates them to differentiate into macrophages [38]. Kazazi et al. showed that during *in vitro* differentiation of monocytes to macrophages, the CD4 membrane antigen is gradually lost, but the mRNA for CD4 and the level of CD4 protein in the cells do not change [31]. This indicates a post-translational mechanism of CD4 downregulation. In contrast to the results obtained by Kazazi et al., significant decrease in CD4 expression during monocyte to macrophage differentiation was not observed by Zhen et al. [38]. An interesting role of CD4 in changing the mechanical properties of the cell membrane was presented in the studies by Bui and Nguyen [39]. The researchers have found that the presence of CD4 in human embryonic kidney 293T cells and Jurkat leukemic cells (clone E6-1) increase the membrane elasticity, while in CD4-negative cells, the membrane is stiffer. The presence and density of CD4 on canine monocytes might be therefore associated whith the differentiation of these cells to macrophages or dendritic cells. It can be hypothesized that the monocyte subsets identified as MHCII^+^CD4^+^ and MHCII^–^CD4^+^ represent cells at a later stage of development and compared to MHCII^+^CD4^-^ monocytes, preferentially differentiate into macrophages. On one hand, during the differentiation of monocytes, their size increases and the shape changes, while in our study it was shown that the cells within both CD4^+^ monocytes subsets are smaller than MHCII^+^CD4^–^ cells. Therefore, further research is needed to evaluate the expression and role of CD4 during maturation of monocytes to macrophages in dogs. In our studies, the phenotypic evaluation concerned only CD14^+^ monocytes. The study of Gibbons et al. was published when our research was ongoing and we have already started a different approach to monocyte cytometric analysis. However, it would be also interesting to evaluate the expression of CD4 on CD14^–^MHCII^+^ monocytes. It should be emphasized that for future studies, there is a need to find a marker that will allow identification of all peripheral blood monocytes in dogs, regardless of the changes in the density of: CD14, CD4 and MHCII surface molecules.

Interesingly, CD4 molecule is also present at high density on canine neutrophils [36]. In our study, a commercially available anti-neutrophil antibody (clone CADO48a) was used to separate neutrophils from monocytes. This labelling also allowed the evaluation of CD4 expression on this leukocyte population, as an additional observation. It has been shown that the small fraction of CADO48a^+^ neutrophils does not express CD4 **(S1 Fig**). Due to the small percentage of these cells among the population of CADO48a+ neutrophils, it can be suggested that they represent not fully differentiated cells, e.g. banded neutrophils. This is also supported by the fact that according to the FSC and SSC parameters CD4^-^ neutrophils are smaller and less granular. However, further research is needed to determine the stage, function and morphology of the CADO48a^+^CD4^–^ neutrophils.

In our research it was also found that both subsets of CD4^+^ monocytes are more effective in the basal production of ROS than predominant subset of MHCII^+^CD4^–^ cells. ROS production by monocytes indicates the development of inflammation in the course of many diseases. Degasperi et al. proved that in obese patients, the production of reactive oxygen species and endoplasmatic reticulum stress in peripheral blood monocytes increased, which demonstrated the proinflammatory activity of these cells and their participation in the development of adipose tissue inflammation [40]. Other authors revealed that in sepsis monocytes show significantly higher ROS production than in healthy controls [41]. Interestingly, the increase in ROS production by monocytes and MDSC has been indicated also as associated with their immunosuppressive potential. In neoplastic diseases, ROS released by monocytic-MDSC contribute to the inhibition of antigen-specific activity of CD8^+^ T cells in cell to cell contact [42]. The *in vitro* model proved that exosomes derived from pancreatic cancer cells promote the acquisition of monocyte with a suppressive phenotype, manifested as decrease in the expression of HLA-DR molecule and an increase in the production of ROS [43]. The increased activity associated with the production of ROS by Mo2 and Mo3 in dogs may therefore indicate their immunomodulatory potential. It is possible that one or both of these monocyte subsets promote inflammatory or neoplastic diseases. To confirm this hypothesis, further research is necessary taking into account the role of these cells in various pathological states. Evaluation of monocyte phenotypic and functional heterogeneity is helpful for future studies, focusing on their activity in inflammation, viral infections or other health disorders, as well as on the identification of remaining small myeloid subsets e.g. monocytic-MDSC.

## Conclusions

Peripheral blood monocytes of dogs vary in the presence of MHCII and CD4, and show different activity regarding the basal production of ROS, that allow to divide them into three different subsets. The higher production of ROS by small subsets of canine monocytes—Mo2 and Mo3, might indicate their immunomodulatory potential. Future research should be focused on assessing changes in their quantitative contribution in certain pathological conditions and determining other differences in their activity.

## Supporting information

S1 Fig Variability of CD11b+CADO48a+ neutrophils in peripheral blood of healthy dogs. The percentages of two neutrophil subsets: CD4-positive (CD4^+^; checkered bar) and CD4-negative (CD4^–^; black bar) in peripheral blood of healthy dogs. (B) Exemplary dot plots presenting flow cytometry gating strategy of neutrophils; first region was set on myeloid cells positive for CD11b (upper left), then CADO48a-positive and CD14^–^negative neutrophils were gated (upper right) and analyzed by the expression of CD4 on CADO48a V500-A vs. CD4 PE-Cy7-A dual fluorescence dot plot (lower right); no specific staining for CD4 was shown when using isotype control (lower left). Graphs presenting differences in (C) size and (D) granularity (according to FSC-H and SSC-H parameter respectively) between CD4^+^ and CD4^–^ neutrophils. Bar graphs of MFI (mean fluorescence intensity) of (E) CD11b and (F) CADO48a expressed on the CD4^+^ and CD4^–^ neutrophils. Data are presented as mean ± SD (n = 13); the significance was determined by Wilcoxon test (**p ≤ 0.01; ***p ≤ 0.001).
(TIF)
