# Supplementary figures and images for: CD4 and MHCII phenotypic variability of peripheral blood monocytes in dogs
Source: PLoS One. 2019 Jul 3;14(7):e0219214. doi: 10.1371/journal.pone.0219214 (PMC6608971; doi:10.1371/journal.pone.0219214)

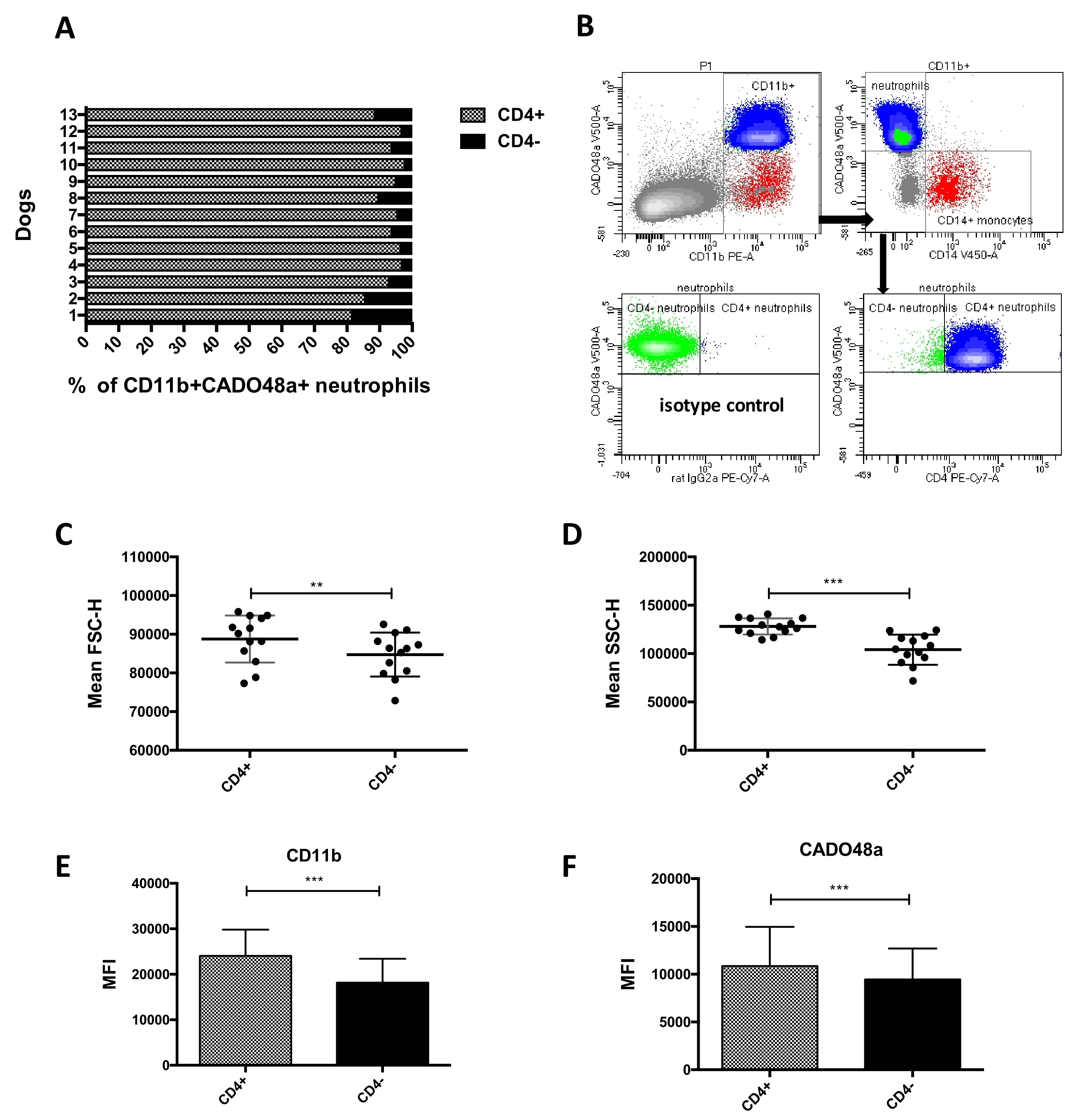

Supplement: S1 Fig — The percentages of two neutrophil subsets: CD4-positive (CD4+; checkered bar) and CD4-negative (CD4–; black bar) in peripheral blood of healthy dogs. (B) Exemplary dot plots presenting flow cytometry gating strategy of neutrophils; first region was set on myeloid cells positive for CD11b (upper left), then CADO48a-positive and CD14–negative neutrophils were gated (upper right) and analyzed by the expression of CD4 on CADO48a V500-A vs. CD4 PE-Cy7-A dual fluorescence dot plot (lower right); no specific staining for CD4 was shown when using isotype control (lower left). Graphs presenting differences in (C) size and (D) granularity (according to FSC-H and SSC-H parameter respectively) between CD4+ and CD4– neutrophils. Bar graphs of MFI (mean fluorescence intensity) of (E) CD11b and (F) CADO48a expressed on the CD4+ and CD4– neutrophils. Data are presented as mean ± SD (n = 13); the significance was determined by Wilcoxon test (**p ≤ 0.01; ***p ≤ 0.001). (TIF) [file pone.0219214.s001.tif]
